# Menstrual cycle inspired latent diffusion model for image augmentation in energy production

**DOI:** 10.1038/s41598-025-99088-4

**Published:** 2025-05-14

**Authors:** Gamal M. Mahmoud, Mostafa Elbaz, Wael Said, Amira A. Elsonbaty

**Affiliations:** 1 Department of Electrical Engineering, Pharos University in Alexandria, Alexandria, Egypt; 2 Department of Computer Science, Faculty of Computers and Informatics, Kafrelsheikh University, Kafrelsheikh, Egypt; 3 Computer Science Department, Faculty of Computers and Informatics, Zagazig University, Zagazig, 44511 Egypt; 4 Communication and Electronics Department, High Institute of Engineering and Technology, New Damietta, 34517 Egypt

**Keywords:** MCI-LDM, Latent diffusion model, Menstrual cycle-inspired metaheuristic, Image augmentation, Energy production, Data processing, Hydrogen energy, Solar energy, Wind energy, Computational science, Computational models

## Abstract

In the energy production domain, image classification is critical for monitoring, diagnostics, and operational optimization tasks. Latent diffusion models (LDMs) have shown potential in generating diverse images during the augmentation process based on text input. However, they are hindered by pixel integrity, texture consistency, and mode collapse. This paper introduces menstrual cycle-inspired latent diffusion model (MCI-LDM), a novel framework that addresses these challenges with three key modifications. First, a menstrual cycle-inspired metaheuristic algorithm is integrated to improve generated images’ pixel integrity and structural coherence. Second, an adaptive attention mechanism is employed to dynamically focus on critical regions during image generation, ensuring that fine details are preserved. Third, a multi-scale feature enhancement module is incorporated to capture global structures and local textures, mitigating mode collapse and enhancing overall image quality. Extensive experiments were conducted on five energy-related datasets, demonstrating the superior performance of MCI-LDM in terms of image augmentation, diversity, and generation accuracy. The results highlight the efficiency of the proposed model, making it a valuable tool for improving image classification and data augmentation in energy sector applications. MCI-LDM outperforms LDM by generating more diverse images, with a higher Inception Score (7.1 vs. 5.4) and a lower Fréchet Inception Distance (22.5 vs. 35.2), indicating better quality and variation. Additionally, MCI-LDM preserves image integrity more effectively, achieving superior PSNR (32.7 dB vs. 28.5 dB) and SSIM (0.92 vs. 0.78).

## Introduction

Energy production is a cornerstone of modern society, providing the essential power needed to run industries, homes, and services globally^1^. Energy generation encompasses various sources, including fossil fuels, renewable sources like solar and wind, and nuclear power^2^. Effective management and optimization of energy systems are critical for improving efficiency, reducing costs, and ensuring sustainability^3,4^. With the growing demand for cleaner and more efficient energy production methods, the sector has increasingly relied on advanced technologies such as artificial intelligence (AI) and machine learning to analyze vast datasets, optimize energy generation, and monitor operational health^5,6^.

In energy production, vast datasets are collected from different stages of the process, including generation, transmission, and distribution. These datasets can contain various forms of information, such as sensor readings, thermal images, satellite data, and operational logs^7–9^. Classification of these datasets is crucial in energy production, particularly for fault detection, predictive maintenance, and anomaly detection^10^. For instance, thermal images of power plants can be classified to detect overheating equipment, while satellite images of solar farms can be used to classify operational efficiency. Machine learning techniques are extensively used to classify such datasets, extracting meaningful insights that drive operational decisions and improvements^11,12^.

Data augmentation is a technique used to increase the size and diversity of datasets, particularly in situations where labeled data is scarce^13,14^. In the energy sector, augmentation is especially useful for enhancing image datasets in anomaly detection, fault classification, and infrastructure monitoring tasks^15,16^. Data augmentation improves model robustness and generalization by creating variations of existing images—such as rotations, flips, or noise injection^17^. Augmentation also allows for the simulation of rare events, such as power failures or equipment malfunctions, enabling machine learning models to be trained on scenarios they might rarely encounter in real-world operations^18,19^. However, traditional augmentation techniques have limitations, particularly in maintaining the fidelity and complexity of images in highly variable energy datasets^20^.

Pixel integrity is fundamental in image generation, especially when generating synthetic images for sensitive tasks such as monitoring energy infrastructure. Pixel integrity refers to the ability of a model to maintain consistency and coherence in the relationships between pixels, preserving the fine details and textures that are critical for accurate image interpretation. In the energy sector, maintaining pixel integrity is particularly important for detecting minute faults or abnormalities in thermal or satellite images. A breakdown in pixel integrity can lead to blurred or unrealistic images, making the augmented data less reliable for training machine learning models. Ensuring each pixel accurately reflects its surrounding context is essential for generating high-quality, usable images^21^.

This paper introduces MCI-LDM (menstrual cycle-inspired latent diffusion model), a novel framework designed to address the challenges associated with LDMs, particularly pixel integrity and mode collapse. Our approach integrates three key modifications to improve the performance of LDMs in generating diverse, high-quality images for energy dataset augmentation. First, we propose a menstrual cycle-inspired metaheuristic algorithm that enhances pixel integrity by assigning adaptive weights to surrounding pixels, ensuring that generated images maintain structural coherence. Second, we introduce an adaptive attention mechanism to prioritize the generation of critical regions, preserving fine details that are essential in energy datasets. Finally, we incorporate a multi-scale feature enhancement module that processes information at multiple resolutions, addressing mode collapse by ensuring the generation of diverse images with both local textures and global structures intact.

**The background and motivation are as follows**: The growing complexity and scale of energy production systems have made it increasingly important to leverage advanced technologies like machine learning and artificial intelligence for monitoring, diagnostics, and optimization. Energy production involves vast datasets from sources such as sensor readings, thermal images, and satellite data, which are critical for fault detection and predictive maintenance tasks. However, these datasets often lack the diversity and quantity needed for robust machine learning model training, making data augmentation essential. Traditional augmentation techniques, while useful, struggle to maintain the fidelity and complexity of images in energy datasets, limiting their effectiveness. Latent diffusion models (LDMs) have emerged as promising tools for generating synthetic images, but they still face significant challenges such as pixel integrity issues, mode collapse, and insufficient detail preservation. This research is motivated by the need to overcome these limitations and enhance LDMs to produce high-quality, diverse images that meet the specific requirements of energy sector applications, ultimately contributing to more accurate and reliable energy system operations.

**The scope and objectives are as follows**: This research uses advanced machine learning models to enhance image augmentation techniques in the energy production domain. Specifically, it aims to address the limitations of existing latent diffusion models (LDMs), such as pixel integrity issues, mode collapse, and inadequate attention mechanisms that affect the quality and reliability of generated images. This study aims to develop a novel menstrual cycle-inspired latent diffusion model (MCI-LDM) that integrates innovative components like a Metaheuristic Algorithm, adaptive attention mechanism, and multi-scale feature enhancement module. These enhancements are designed to improve pixel integrity, capture fine details, and ensure structural coherence in generated images. The ultimate goal is to create high-quality, diverse synthetic images that can be effectively used for machine learning tasks in energy applications, such as fault detection, predictive maintenance, and operational optimization, thereby contributing to more efficient and reliable energy systems.

The contributions of this paper:

- Developed a novel menstrual cycle-inspired latent diffusion model (MCI-LDM) that addresses key challenges in image generation for energy dataset augmentation, particularly pixel integrity and mode collapse.
- Proposed a unique menstrual cycle-inspired metaheuristic algorithm to enhance pixel integrity by assigning adaptive weights to surrounding pixels, ensuring structural coherence in generated images.
- Introduced an adaptive attention mechanism that dynamically prioritizes the generation of critical regions in images, preserving fine details that are crucial in energy datasets.
- Implemented a multi-scale feature enhancement module to capture global structures and local textures, addressing the problem of mode collapse and improving image diversity.
- Conducted extensive experiments on five energy-related datasets, demonstrating the efficiency of the proposed model in generating high-quality, diverse images for data augmentation.
- The results showed a significant improvement in the augmentation process, leading to enhanced machine learning model performance for classification tasks in the energy production domain.
- Proven the generalizability and efficiency of the MCI-LDM model in handling diverse energy datasets, making it a robust solution for real-world applications in energy production.

The organization of this paper is structured to provide a comprehensive understanding of the proposed approach and its impact on the energy production domain. Section “Related work” reviews the related work, highlighting existing challenges and gaps in latent diffusion models (LDMs) and their application in image augmentation for energy systems. Section “Methodology” details the methodology, introducing the menstrual cycle-inspired latent diffusion model (MCI-LDM) and its key components, including the Metaheuristic Algorithm, adaptive attention mechanism, and multi-scale feature enhancement module. Section “Results and discussion” presents the results and discussion, showcasing the performance of MCI-LDM through extensive experiments on various energy-related datasets, comparing its effectiveness with existing models, and analyzing its impact on image augmentation quality. Finally, Section “Conclusion and future work” concludes the paper, summarizing the key findings and contributions, and outlines future directions for enhancing LDMs to improve their applicability in critical energy sector tasks.

## Related work

Latent diffusion models (LDMs) have emerged as a powerful tool in image augmentation, particularly in the energy sector, where generating high-quality synthetic images can significantly enhance machine learning model performance. Rombach et al.^22^ introduced LDMs as a method that uses latent space diffusion processes to create diverse images from text inputs, making them highly valuable for data augmentation. These models are particularly useful in scenarios with limited labeled data, as they can generate a wide range of realistic images to improve model training. However, LDMs often face pixel integrity challenges, where pixel relationship inconsistencies can result in images lacking the fine details necessary for sensitive applications such as energy infrastructure monitoring.

Text-to-image generation using LDMs has been explored in multiple industrial contexts, with several studies emphasizing their adaptability and control over the generated content. Saharia et al.^23^ introduced Imagen, an advanced text-to-image diffusion model that outperformed traditional generative approaches regarding fidelity and diversity. However, when applied to specific sectors such as energy, maintaining the pixel integrity of generated images becomes critical. The model’s reliance on latent space transformations can sometimes lead to pixel inconsistencies, particularly in detailed regions, impacting the reliability of the images for practical applications.

Nichol et al.^24^ also highlighted the issue of pixel integrity in LDMs in their work on improved denoising diffusion probabilistic models (DDPMs). While LDMs can produce visually appealing images, maintaining coherent relationships between pixels remains a significant challenge, especially when generating complex textures or fine details required in energy datasets. This pixel inconsistency can lead to artifacts or blurring, which diminishes the usefulness of the generated images for tasks such as identifying subtle faults in thermal imaging or satellite monitoring of energy infrastructure.

The limitations of LDMs in preserving pixel integrity are not only a technical challenge but also pose practical constraints in their application. Dhariwal and Nichol^25^ demonstrated that although improved diffusion models could enhance image quality, they often failed to maintain fine detail across large-scale image generation tasks. In energy applications, this can result in synthetic images that do not accurately reflect the physical properties needed for precise fault detection and predictive maintenance, limiting the effectiveness of the augmented datasets.

Another notable study by Ho et al.^26^ discussed incorporating attention-based mechanisms into diffusion models to enhance image fidelity and reduce inconsistencies. While these improvements have helped LDMs focus on critical regions during image synthesis, they are often insufficient for energy-related applications that demand high precision in pixel-level accuracy. Though visually coherent, the generated images may still lack the fine texture details needed for accurate machine learning model training, particularly in detecting minor anomalies such as small cracks or material deformations.

Recent advancements in generative modeling highlighted the efficacy of diffusion models, particularly through the introduction of the Swin-Transformer-based Latent Diffusion Model (ST-LDM) by Xue et al.^27^. The introduced framework used a universal integration capability with existing latent diffusion models while incorporating a training-free backward guidance mechanism. Central to ST-LDM was a global-perceptual autoencoder that utilized adaptable compression scales and hierarchical visual features, enhancing the model’s representation capacity. Additionally, a deformable multimodal transformer facilitated the generation of region-wise guidance critical for the denoising process. Notably, ST-LDM addressed the limitations of traditional attention mechanisms that predominantly focused on existing visual features by implementing deformable feature alignment, which allowed for hierarchical refinement of spatial positioning and the integration of multi-scale visual and linguistic information. Empirical evaluations demonstrated that ST-LDM significantly enhanced attention localization while preserving the generative capabilities intrinsic to diffusion models, establishing a foundational reference for future research aimed at optimizing attention mechanisms in generative frameworks.

Gao et al.^28^ introduced the Disruptive Text-Image Alignment (DTIA) framework, which addresses the vulnerabilities in existing text-image alignment methods that stem from an overfitting of textual inputs to noise, leading to inaccurate face information matching. The authors designed a Text-Image Mis-Match Attack framework aimed at disrupting the model’s learning of associations between input faces and corresponding texts, thereby mitigating unnecessary computational burdens. Additionally, they investigated the impact of timestep selection in diffusion models on the effectiveness of adversarial attacks and proposed a step schedule strategy to enhance algorithmic efficiency. Extensive experiments conducted on facial benchmark datasets demonstrated that the DTIA framework not only effectively disrupted personalized generation models but also significantly improved overall model efficiency, establishing a critical advancement in the field of text-image alignment and adversarial modeling.

LDMs face several challenges that can hinder their performance and effectiveness in generating high-quality images. One significant issue is the potential for mode collapse, where the model generates a limited variety of outputs, failing to capture the full diversity of the training data. This phenomenon can lead to repetitive and less interesting images, undermining the model’s utility in creative applications. Additionally, maintaining pixel integrity between neighboring pixels poses a challenge, as slight discrepancies can result in visual artifacts that detract from the overall quality of generated images^29,30^.

In the area of maintaining the integrity between pixels in the generated images, many meta-heuristics algorithm has been used to solve this issue. Metaheuristic algorithms play a crucial role in maintaining pixel integrity in image generation by optimizing pixel relationships and enhancing overall image quality. Various methods can be employed within this framework, such as Genetic Algorithms (GA)^31^, which simulate natural selection to evolve pixel arrangements for better coherence and detail preservation. Particle Swarm Optimization (PSO)^32^ can also be utilized to adjust pixel values based on neighboring pixel interactions, ensuring smoother transitions and reducing artifacts. Several new and emerging metaheuristic algorithms can effectively maintain pixel integrity in image generation^33,34^. The Cuckoo Search Algorithm (CSA), inspired by the brood parasitism of cuckoos, uses a combination of random walks and local search to explore diverse pixel configurations while focusing on high-quality solutions. The Grey Wolf Optimizer (GWO) mimics the social hunting behavior of grey wolves, utilizing a hierarchical structure to balance exploration and exploitation in the pixel space. Similarly, the Salp Swarm Algorithm (SSA), inspired by the movement patterns of salps, simulates their behavior to ensure smooth transitions and maintain image detail. The Whale Optimization Algorithm (WOA), which emulates the hunting strategies of humpback whales, employs a spiral updating mechanism to enhance pixel relationships and reduce artifacts. Another innovative method is the Ant Lion Optimizer (ALO), which models the hunting behavior of ant lions to trap and optimize pixel arrangements for high-fidelity results. The Sine Cosine Algorithm (SCA) utilizes oscillatory functions to adjust pixel values while preserving fine details. The Multi-Verse Optimizer (MVO) explores pixel relationships by simulating interactions among multiple universes, leading to diverse image generation. The Grasshopper Optimization Algorithm (GOA) draws on the swarming behavior of grasshoppers, optimizing pixel configurations through collective behavior. The Chimera Algorithm (CA) also used to blend multiple optimization techniques to adaptively enhance pixel integrity, ensuring high-quality image generation. Together, these algorithms represent a robust set of tools for tackling pixel integrity challenges in various applications^35–37^.

### Research gaps

- Current LDMs struggle with maintaining coherent pixel relationships, leading to artifacts and blurred textures. This is problematic for applications requiring high-fidelity images, such as energy infrastructure monitoring, where fine details are critical for detecting faults.
- LDMs often suffer from mode collapse, which results in limited diversity in generated images. This restricts the representation of various scenarios, crucial for comprehensive model training in energy applications.
- Although attention mechanisms have been integrated into LDMs to focus on critical image regions, they still fail to maintain the fine detail required in sectors like energy, where pixel-level accuracy is necessary for reliable model training.
- LDMs are highly adaptable for generating images from text inputs, but they often produce images with pixel inconsistencies, especially when dealing with complex textures in energy datasets.
- To address pixel integrity and mode collapse, further development of multi-scale feature enhancement modules that can capture both global structures and local textures is needed.
- LDMs are limited in their ability to generate images that accurately simulate rare but critical events like equipment failures, which are essential for training models in predictive maintenance and fault detection.

## Methodology

The methodology of this study involves developing and applying the menstrual cycle-inspired latent diffusion model (MCI-LDM) to enhance image augmentation in energy-related applications. The process begins with utilizing several energy datasets, each containing images for tasks such as fault detection and system monitoring. Although initial augmentation methods such as rotations, zooming, and noise injection are applied, these datasets still suffer from limited diversity, particularly in capturing rare and critical events. To address these issues, MCI-LDM incorporates several innovative components, including the menstrual cycle-inspired metaheuristic algorithm (MCMHA), adaptive attention mechanism, and Skip Connection techniques.

Image generation starts by creating latent representations from Gaussian noise and encoding energy-related text into embeddings, ensuring alignment between textual input and generated images. These latent representations are then processed using a latent U-Net model, which conditions the latent space based on the input text. The model integrates Skip Connections to retain essential features and prevent information loss during the transformation process, enhancing the images’ overall coherence and structural fidelity. The conditioned latents undergo iterative refinement through a scheduler algorithm, progressively transforming noisy latent into structured and high-quality images.

An adaptive attention mechanism is employed within the U-Net architecture to dynamically focus on critical regions of the images, preserving fine details that are crucial for energy applications, such as identifying faults in complex environments. This attention mechanism ensures that the generated images accurately capture global structures and intricate local textures, addressing common challenges like pixel inconsistency and mode collapse.

After refinement, the latent are decoded into images using a Variational Autoencoder (VAE), followed by a pixel conversion process to ensure pixel-level accuracy. The MCMHA is then applied to optimize pixel integrity by simulating biological cycles, which systematically adjust and enhance pixel relationships through multi-phase optimization, reducing artifacts and improving overall image quality. If the generated images pass the integrity checks, they are incorporated into the dataset for further augmentation; if not, the process reverts to earlier stages for additional refinement. Combining MCMHA, adaptive attention mechanism, and Skip Connections, this comprehensive approach ensures that the generated images are highly quality, diverse, and suitable for practical energy sector applications, such as infrastructure monitoring and fault detection.

Pseudocode (1) shows the methodology’s sequential steps, emphasizing the integration of Skip Connections, adaptive attention mechanism, and MCMHA for iterative refinement and quality assurance in image generation. Each step ensures that the generated images maintain structural integrity, pixel accuracy, and detail preservation, making them suitable for energy-related tasks like fault detection and monitoring.

**Pseudocode 1:**
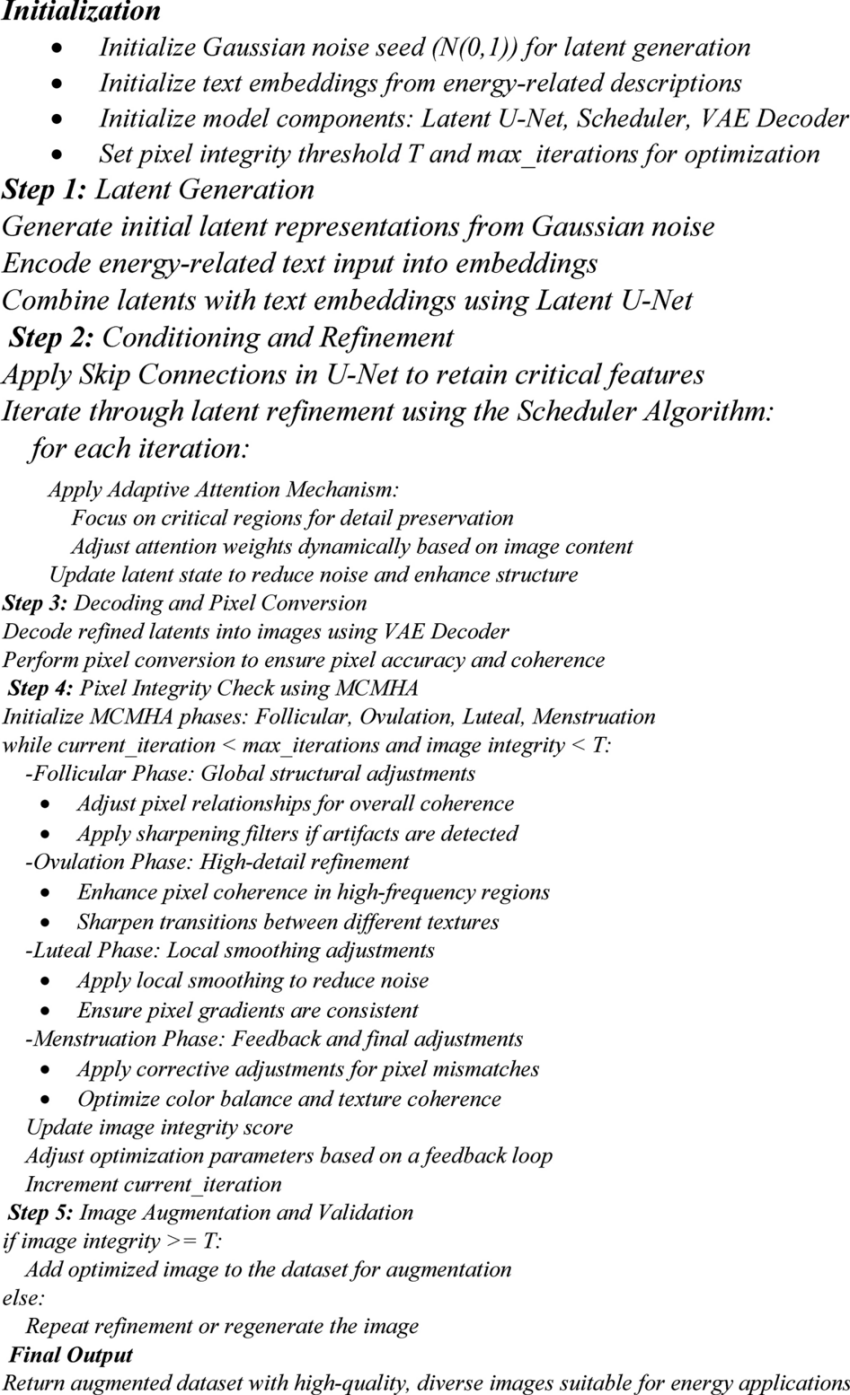
Pseudocode for MCI-LDM Methodology.

### Datasets

The paper uses 5 different energy and electric dataset to prove the efficiency of the augmentation and diversity process using the novel MCI-LDMs.

**Fig. 1:**
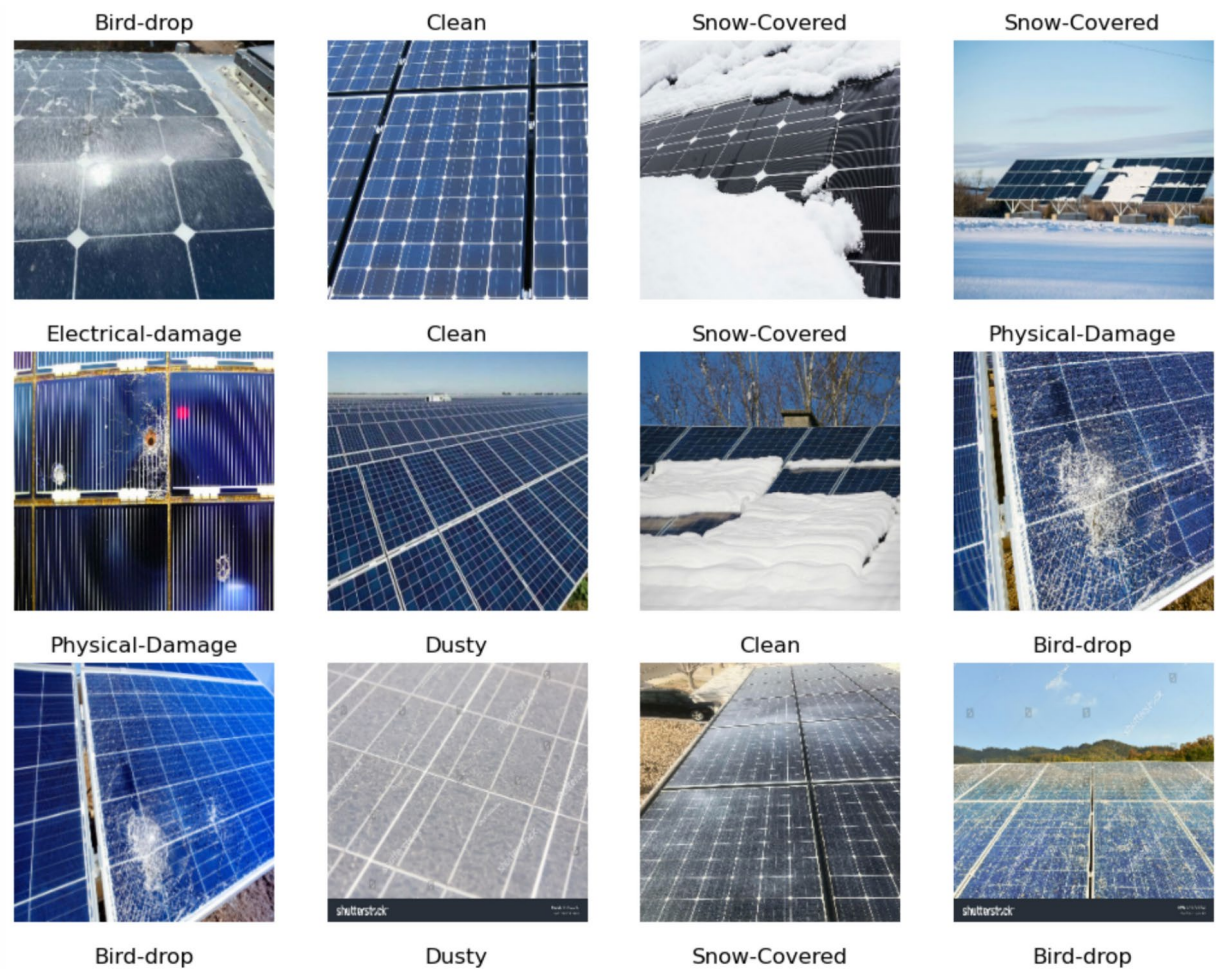
Solar panel for power generation dataset exploration.

The Energy Fault Detection^38^ dataset comprises 15,000 images aimed at identifying various faults in solar panel cells, serving as a vital resource for monitoring and maintaining solar energy systems. It includes several labels related to common issues, such as dust accumulation, snow cover, bird droppings, cracks, discoloration, and electrical faults. Representative sample images from the dataset illustrate these faults, with detailed descriptions explaining their usage in training machine learning models for accurate fault detection. For instance, images showing dust accumulation can help algorithms learn to recognize efficiency losses due to debris, while those depicting cracks can assist in identifying structural integrity issues. However, the dataset has notable limitations, particularly in its lack of diversity; it may not adequately capture a wide range of environmental conditions or rare fault cases, such as extreme weather impacts or unusual debris types. This limitation makes it difficult to model critical events that could significantly affect solar panel performance. Additionally, the dataset could benefit from enhanced data augmentation techniques to simulate various scenarios, such as different lighting conditions or angles of dirt accumulation. Addressing these diversity issues and enhancing the dataset’s robustness is crucial for improving its utility in developing effective monitoring and maintenance strategies for solar panels. Figure 1 shows the examples of solar panel exploration with different labels.

The Solar Farm Monitoring dataset^39^ consists of 12,500 images designed to support the monitoring and maintenance of solar farms, providing essential insights for optimizing solar energy production. The dataset includes labels for various conditions, such as operational efficiency, panel defects, and environmental impacts. Representative sample images illustrate these conditions, with detailed descriptions explaining their application in training machine learning models for effective monitoring. For example, images showcasing operational efficiency.

The Wind Turbine Inspection dataset^40^ comprises 10,000 images designed to facilitate the identification of mechanical faults in wind turbines, making it an essential resource for ensuring the safety and efficiency of wind energy systems. The dataset includes labels for various issues such as blade cracks, corrosion, loose components, and other mechanical failures. Representative sample images illustrate these faults, with detailed descriptions provided to demonstrate how they are utilized in training machine learning models for effective fault detection. For example, images depicting blade cracks can help algorithms learn to identify structural vulnerabilities, while those showing corrosion can aid in assessing material degradation. However, the dataset faces significant challenges in capturing the variety of mechanical faults and environmental factors necessary for comprehensive inspections, particularly under extreme conditions such as high winds, heavy rain, or snow. This limitation restricts the dataset’s ability to model critical events that may lead to turbine failures. Additionally, it may not adequately represent rare fault cases or the diverse operational environments in which wind turbines function. Enhanced data augmentation techniques could improve the dataset by simulating various weather conditions and mechanical scenarios. Addressing these diversity issues and enhancing the dataset’s robustness is crucial for optimizing inspection processes and ensuring the reliability of wind turbine operations.

The Smart Grid Anomaly Detection^41^ dataset consists of 8,000 images designed to support the identification of anomalies and abnormal events within electrical grids, making it a vital resource for enhancing grid reliability and performance. It includes labels for various scenarios, such as power surges, equipment malfunctions, and unusual load patterns. Representative sample images illustrate these anomalies, with detailed descriptions explaining their application in training machine learning models for effective detection. For instance, images showcasing power surges can aid algorithms in recognizing patterns that precede failures, while those depicting equipment malfunctions can help in diagnosing system vulnerabilities. However, the dataset is limited by the rarity of grid anomalies, making it challenging to model less frequent scenarios effectively. This limitation restricts the dataset’s ability to prepare algorithms for critical events that could significantly impact grid stability. Additionally, the dataset might benefit from enhanced data augmentation techniques to simulate diverse operational conditions and rare anomalies, addressing these diversity challenges is crucial for improving the dataset’s utility in smart grid management.

Similarly, the Hydroelectric Plant Monitoring dataset^42^ contains 9,500 images aimed at facilitating the monitoring of hydroelectric plants to ensure optimal performance and safety. It includes labels for various conditions, such as water flow levels, structural integrity, and equipment status. Representative sample images illustrate these conditions, with detailed descriptions about how they are used in training machine learning models for effective monitoring. For example, images depicting water flow variations can assist algorithms in identifying optimal operating conditions, while those showing structural issues can help in assessing risks to operational safety. However, the dataset suffers from limited variation in water flow and structural issues, which restricts its ability to capture rare operational failures. This limitation makes it difficult to model critical scenarios that may lead to significant operational risks. To enhance the dataset’s robustness, implementing data augmentation techniques that simulate different water flow conditions and structural scenarios could be beneficial. Addressing these diversity issues is essential for improving the dataset’s effectiveness in monitoring hydroelectric plant operations.

Table 1 provides a comprehensive overview of the datasets utilized by the methodology, highlighting key characteristics such as the number of images, their intended use, the applied augmentation processes, and limitations. Each dataset is employed in energy-related image classification tasks, including fault detection and system monitoring. Despite the application of various augmentation techniques like rotations, zooming, and noise injection, all datasets are marked as limited, primarily due to insufficient diversity in the images.

**Table 1:** Datasets used by the methodology.

Dataset name	Number of images	Used for	Limited dataset (true/false)	Reason for limitation
Energy Fault Detection	15,000	Fault detection in power generation systems in solar panels	True	Limited diversity in environmental conditions; insufficient for rare fault cases
Solar Farm Monitoring	12,500	Solar panel condition and efficiency assessment	True	Insufficient for capturing rare panel defects under different weather and lighting conditions
Wind Turbine Inspection	10,000	Fault detection and maintenance of wind turbines	True	Limited variety of mechanical faults and angles, especially under extreme weather conditions
Smart Grid Anomaly Detection	8000	Anomaly detection in smart grid systems	True	Limited examples of anomalies and abnormal grid events, especially rare occurrences like power surges
Hydroelectric Plant Monitoring	9500	Monitoring the structural health of hydroelectric plants	True	Limited variation in water flow and structural issues, insufficient for rare structural failures and anomalies

### Image generation using LDM with menstrual cycle-inspired meta heuristic

**Fig. 2:**
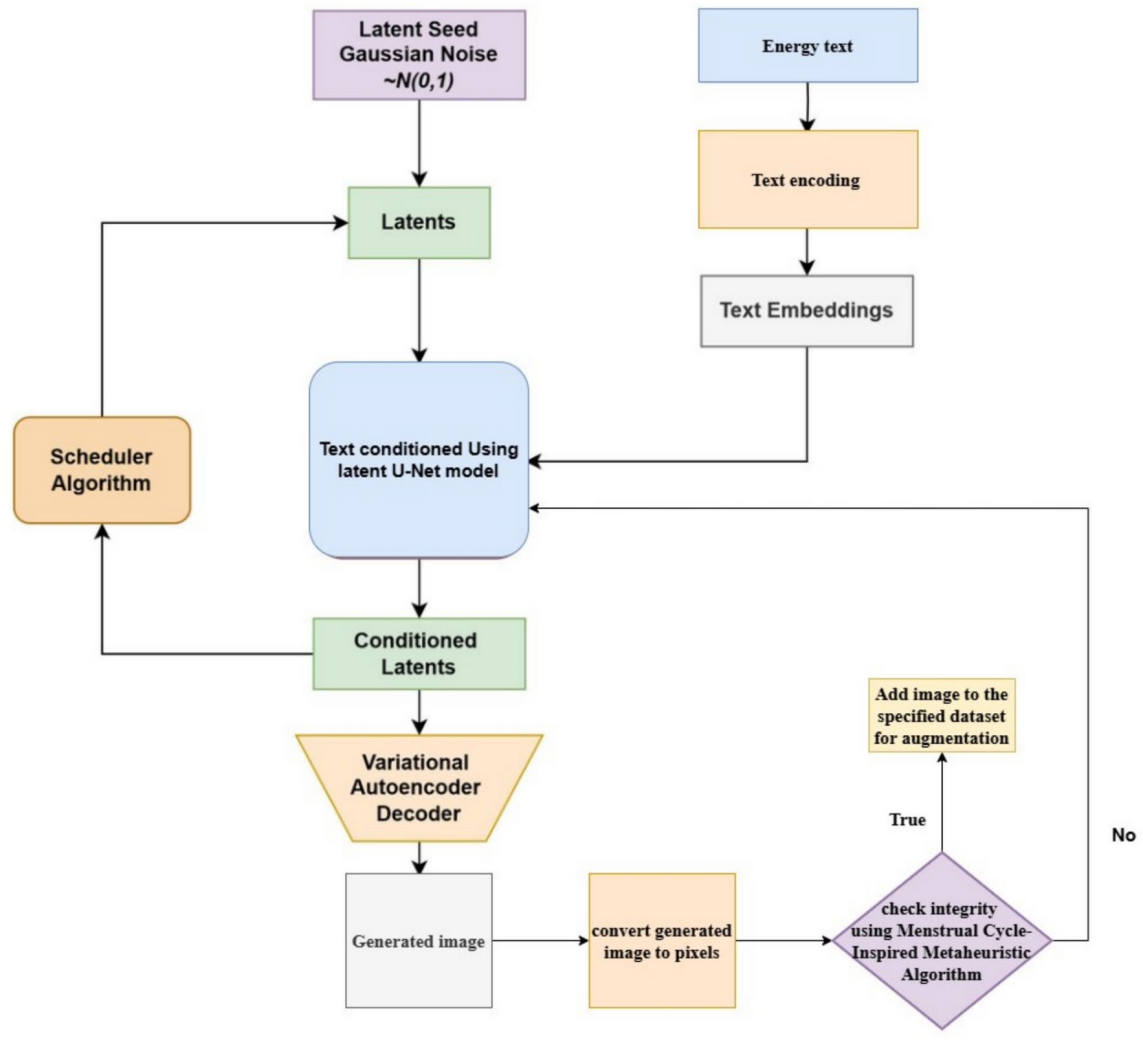
Flow chart of LDM with menstrual cycle inspired meta-heuristic.

Figure 2 illustrates the detailed workflow of a latent diffusion model (LDM) enhanced with a menstrual cycle-inspired metaheuristic algorithm, specifically designed for image generation and augmentation in energy-related applications. The process begins with the generation of a latent seed from Gaussian noise, denoted by N(0, 1). This random noise forms the basis for generating latents, which serve as the foundation for the image creation process in the diffusion model. Energy-related text is encoded using a text encoding mechanism to create text embeddings. These embeddings capture the semantic content of the energy-related input, ensuring that the generated images align with the textual description. The generated latents are combined with the text embeddings in the next step using a latent U-Net model. This model is responsible for conditioning the latent space based on the textual input, thereby guiding the image generation in accordance with the meaning derived from the energy-related text. The U-Net architecture ensures that the features from the text are effectively merged into the latent representation. Once the latents are conditioned, they are iteratively refined using a scheduler algorithm. This algorithm controls the progressive denoising of the latent space, gradually transforming the noisy latents into more structured and coherent representations that correspond to a high-quality image. The scheduler is crucial in refining the latents, ensuring they evolve into meaningful and accurate representations. After the refinement, the latents are passed through a Variational Autoencoder (VAE) Decoder, transforming them into actual images. This decoding process takes the abstract latent representation and converts it into a pixel-based image that can be visually interpreted. Once the image is generated, it undergoes a pixel conversion process to ensure pixel accuracy and coherence. Following pixel conversion, the generated image is subjected to an integrity check using the menstrual cycle-inspired metaheuristic algorithm. This algorithm likely mimics natural cycles to optimize and ensure that the generated image maintains pixel integrity, a critical factor for energy-related applications where detail and accuracy are essential. The algorithm checks whether the image is of sufficient quality, particularly regarding pixel-level details necessary for fault detection in energy systems. If the image passes the integrity check, it is added to the specified dataset for augmentation, expanding the diversity and volume of training data for machine learning models. In cases where the integrity checks fail, the system reverts to earlier stages of the process to regenerate or refine the image further. This workflow demonstrates a robust process of combining a latent diffusion model with a metaheuristic algorithm to ensure high-quality synthetic images tailored to the energy sector. Text conditioning and iterative refinement ensure that the images are semantically accurate and of high pixel integrity, making them suitable for practical applications such as energy infrastructure monitoring and fault detection.

### Menstrual cycle-inspired meta heuristic

The menstrual cycle-inspired metaheuristic algorithm (MCMHA) is a novel optimization technique inspired by the natural biological processes of the menstrual cycle. Like other metaheuristics such as Genetic Algorithms (GA) or Particle Swarm Optimization (PSO), MCMHA is designed to simulate biological phenomena, but with a specific focus on the cyclical hormonal and physiological changes that occur in women. This cycle-based inspiration can be adapted into computational optimization for various tasks, including maintaining the pixel integrity in image generation with latent diffusion models (LDMs). When applied to LDMs, the MCMHA acts as a post-processing optimization phase that improves the consistency and quality of the generated images by checking for pixel-level coherence. The cycle-inspired approach can optimize the interaction between adjacent pixels to resemble biological patterns, allowing for smoother transitions, fewer artifacts, and improved structural integrity in generated images.

In essence, LDMs sometimes struggle with maintaining the fine details at the pixel level, which can result in blurring, distortion, or pixel artifacts, especially in regions requiring high accuracy. The MCMHA helps address this by implementing a series of optimization steps that “correct” or adjust the image in line with natural cycles, which are typically broken down into phases. Each phase mimics a different hormonal state in the menstrual cycle, targeting specific areas of the generated image to focus on refinement and improvement in pixel-level coherence.

#### How MCMHA enhances pixel integrity in LDMs

1. *Cyclic Optimization*: The algorithm simulates a biological cycle, where each phase targets different parts of the image or focuses on different pixel-level relationships. In the menstrual cycle, these phases are characterized by fluctuating hormones, and in the MCMHA, these correspond to different optimization strategies. For example, some phases prioritize enhancing edge detection, while others focus on noise reduction or texture coherence.
2. *Multi-Phase Adjustments*: Just as the menstrual cycle involves different stages (follicular phase, ovulation, luteal phase, etc.), the MCMHA can employ different sub-algorithms or techniques in each phase. This multi-phase approach ensures that pixel integrity is addressed from various angles. For instance, during an “ovulation” phase, the algorithm might introduce global pixel-level adjustments to sharpen image resolution. In contrast, in the “luteal” phase, it focuses on fine-tuning local pixel coherence and smoothing transitions between high-frequency regions.
3. *Hormonal-Inspired Feedback Loops*: In biological terms, the menstrual cycle is regulated by feedback mechanisms involving estrogen, progesterone, and other hormones. Similarly, the MCMHA employs feedback loops where the output of one phase informs the adjustments in the next, allowing for continuous refinement. For example, if pixel artifacts are detected in a high-frequency region during the first phase, the second phase might prioritize corrective measures. Pseudocode (2) shows the steps of **MCMHA**

**Pseudocode 2:**
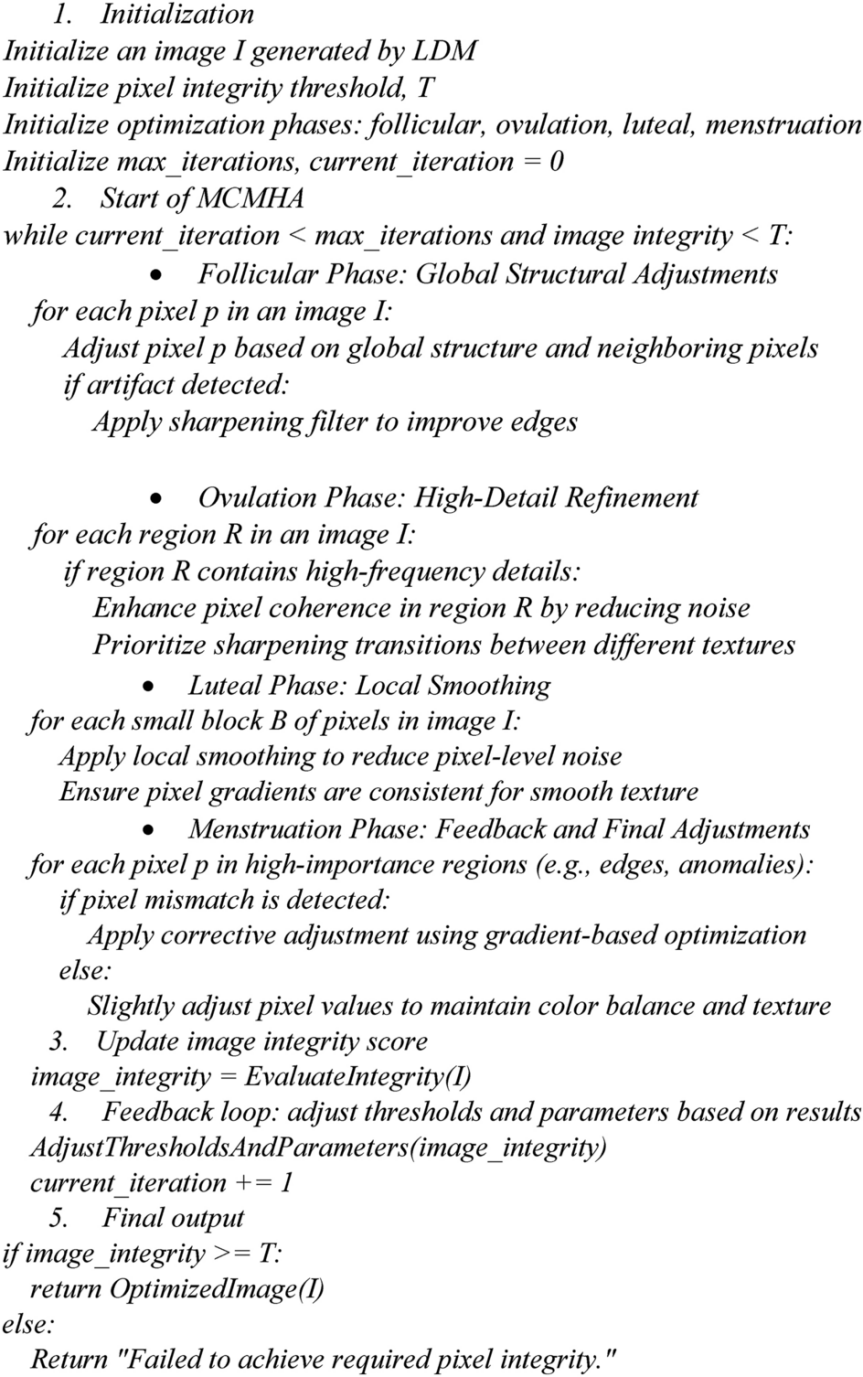
MCMHA

One significant issue is the tendency of algorithms like Genetic Algorithms (GA) and Simulated Annealing (SA) to get trapped in local optima due to their exploration strategies. MCMHA mitigates this by employing a multi-phase approach that enhances global search capabilities. Additionally, algorithms such as Particle Swarm Optimization (PSO) often rely on static parameters, limiting adaptability. In contrast, MCMHA’s dynamic strategy adjusts based on the optimization phase, allowing for more flexible exploration and exploitation. Moreover, some methods, like the Ant Lion Optimizer (ALO), may lack diversity in their search mechanisms, but MCMHA promotes diversity in pixel arrangements through its varied strategies. It also excels in navigating complex image landscapes, where traditional algorithms like Differential Evolution (DE) may struggle, thanks to its adaptive refinement of solutions. The MCMHA incorporates temporal dynamics, mimicking natural cycles to maintain pixel integrity over time, which many traditional algorithms overlook. Lastly, while many algorithms focus on single-objective optimization, MCMHA can inherently manage multiple objectives by strategically alternating between different goals throughout its phases. In addressing these challenges, MCMHA offers a robust alternative that enhances pixel integrity and overall image quality, particularly in complex image generation tasks.

### Modified skip connection at MCI-LDM

The modified Skip Connections in the menstrual cycle-inspired latent diffusion model (MCI-LDM) play a crucial role in enhancing the image generation process by retaining essential features during the transformation stages, thereby preventing information loss that often occurs in deep neural networks. In traditional U-Net architectures, Skip Connections are used to link feature maps from the encoding layers directly to corresponding decoding layers, allowing the model to combine low-level details from earlier layers with high-level abstractions. The modified Skip Connections in MCI-LDM are designed to transfer spatial information and selectively integrate feature maps that are most relevant to preserving pixel integrity and structural coherence, addressing the common challenges of pixel inconsistency and mode collapse. Mathematically, the modified Skip Connection can be represented using Eq. (1).

1 $$F_{skip} = \alpha .F_{encode} + \beta .F_{decode }$$

where ${F}_{encode}$​ represents the feature map from the encoding layer; ${F}_{decode}$ is the feature map from the decoding layer and ${F}_{skip}$ is the feature map of the skip connection. α and β are learnable coefficients that dynamically adjust the contribution of each feature map. The parameters α and β are optimized during training, allowing the Skip Connection to balance low-level spatial information and high-level contextual understanding adaptively. This modified approach ensures that the detailed textures from earlier layers are effectively combined with the overall structural information from deeper layers, enhancing the image’s fidelity and ensuring accurate representation of critical regions.

An adaptive gating mechanism is introduced within the Skip Connection, defined by Eq. (2) where σ denotes the sigmoid function, ${W}_{g}$ is the weight matrix, and ${b}_{g}$ is the bias term. This gating mechanism selectively controls the flow of information between the connected layers, further refining the Skip Connection’s output to focus on preserving the most relevant features. By incorporating these modified Skip Connections, MCI-LDM effectively mitigates the risk of information degradation, thereby improving the quality and coherence of the generated images, particularly in applications where pixel-level accuracy is critical, such as energy infrastructure monitoring and fault detection.

2 $$G = \sigma \left( {W_{g} \cdot \left[ {F_{encode} ,F_{decode} } \right] + b_{g} } \right)$$

### Adaptive Mechanism in MCI-LDM

The adaptive attention mechanism in the menstrual cycle-inspired latent diffusion model (MCI-LDM) plays a pivotal role in enhancing the quality of generated images by dynamically focusing on critical regions during the image synthesis process. This mechanism allows the model to prioritize areas of an image that require greater detail, such as fine textures and complex structures, which are essential for applications like fault detection in energy systems. Unlike conventional attention mechanisms that apply static weights, the adaptive attention mechanism in MCI-LDM continuously adjusts its focus based on the evolving characteristics of the image during generation, ensuring that key features are preserved and highlighted. Mathematically, Eq. (3) can represent the adaptive attention mechanism.

3 $$A_{adaptive} = softmax\left( {\frac{{QK^{T} }}{{\sqrt {d_{k} } }}} \right) \cdot V$$

where Q represents the query, K represents the key, and V represents the value, which are projected representations of the input features, and ${d}_{k}$ is the dimensionality of the Key vectors. The SoftMax function computes the attention weights, allowing the model to concentrate on specific image parts. The adaptive nature comes from dynamically updating Q, K, and V at each stage of the generation process, guided by feedback from the ongoing image synthesis. This adaptive feedback loop ensures that attention is continuously re-calibrated to maintain coherence and fidelity in areas with fine details. In the attention mechanism, inputs are transformed into three distinct representations: queries, keys, and values. The query vector (Q) represents the item that is being focused on, while the key vector (K) corresponds to items that are being compared against the query. The value vector (V) holds the actual information that is retrieved based on the attention scores between the queries and keys. This triplet structure allows the model to flexibly weigh different inputs and retrieve relevant information, enabling more nuanced understanding and processing of the data. The dot product of the query and key matrices, represented as ${QK}^{T}$, computes a score that indicates the relevance of each key to the current query. This score quantifies how similar the query is to each key. The scaling factor $\sqrt{{d}_{k}}$ serves to mitigate the effects of large dot product values. As the dimensionality of the keys increases, the dot products tend to grow larger, which can lead to issues such as vanishing gradients during training.

Additionally, an adaptive weighting factor γ is introduced to modulate the impact of attention across different regions using Eq. (4) where the $F_{latent}$ represents a weighted combination of two distinct components: the adaptive attention output Adaptive $A_{adaptive}$ and the latent feature representation the γ is a scalar weight that controls the contribution of the adaptive attention output to the final output. The term $A_{adaptive}$ reflects the contextually relevant information retrieved from the attention mechanism, which dynamically focuses on the most pertinent aspects of the input. In contrast, ${F}_{latent}$ encompasses the underlying features extracted from the input data, capturing essential patterns that may not be immediately evident. By combining these two components, the equation allows the model to blend contextual information with foundational features

4 $$F_{output} = \gamma \cdot A_{adaptive} + \left( {1 - \gamma } \right) \cdot F_{latent}$$

Through this adaptive attention mechanism, MCI-LDM enhances its ability to generate high-quality, detailed images, effectively capturing global patterns and localized intricacies crucial in energy-related applications. Thus, it significantly improves the reliability and precision of image-based diagnostics and monitoring tasks.

## Results and discussion

In the experiment, the menstrual cycle-inspired latent diffusion model (MCI-LDM) was compared against the traditional Latent Diffusion Model (LDM) using a variety of metrics to assess the quality of generated images in terms of diversity and integrity. To evaluate the diversity of generated images, metrics such as the Inception Score (IS)^43^, Fréchet Inception Distance (FID)^44^, Perceptual Path Length (PPL)^45^, and Diversity Score were utilized. These metrics helped assess the variation, richness, and range of images produced, focusing on ensuring that MCI-LDM explored the latent space more comprehensively compared to LDM, thereby mitigating mode collapse and enhancing diversity.

To measure the integrity of the generated images, Reconstruction Error using Peak Signal-to-Noise Ratio (PSNR)^46^ and the Structural Similarity Index (SSIM)^47^ were employed. These metrics focused on preserving the generated images’ pixel-level details and visual coherence. PSNR quantified the accuracy of the reconstructed images, while SSIM measured the structural similarity between the real and generated images.

Following the image generation, a classification validation step was performed further to evaluate the impact of MCI-LDM on downstream tasks. Several deep learning models were employed, including Vision Transformer (ViT)^48^, EfficientNetV2^49^, ResNet50^50^, DenseNet121^51^, and MobileNetV3^52^, across multiple datasets: Energy Fault Detection, Solar Farm Monitoring, Wind Turbine Inspection, Smart Grid Anomaly Detection, and Hydroelectric Plant Monitoring. These models were trained before and after applying the MCI-LDM-generated images, and their performance was validated using standard classification metrics: accuracy, precision, recall, F1-score, and area under the curve (AUC). This validation step ensured that the MCI-LDM generated more diverse and accurate images and improved classification model performance, leading to better generalization and accuracy in downstream tasks.

### Diversity of generated images measurement

**Fig. 3:**
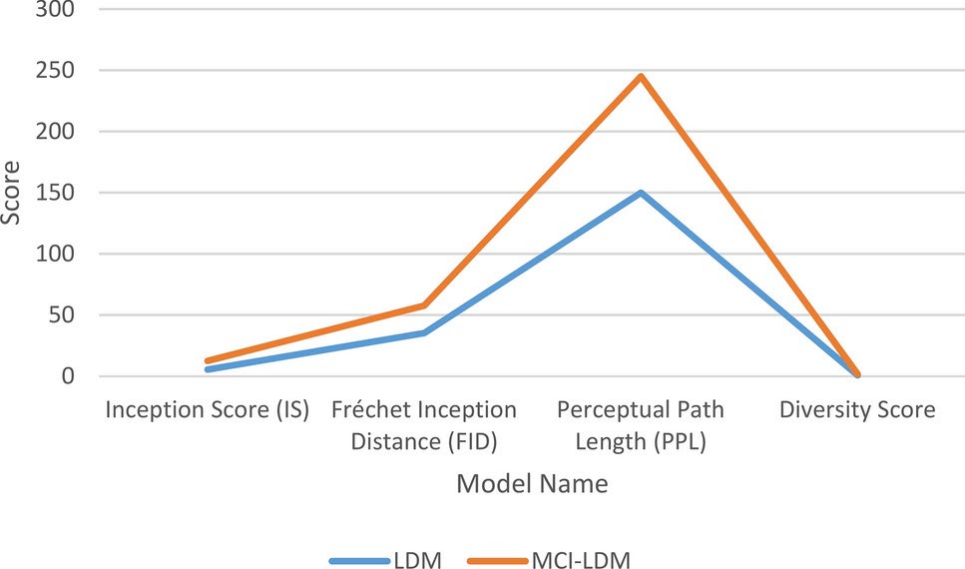
Comparison between LDM and MCI-LDM in terms of diversity.

Our methodology employed several key metrics to evaluate and compare the diversity of images generated by the latent diffusion model (LDM) and our proposed menstrual cycle-inspired latent diffusion model (MCI-LDM). Specifically, we used the Inception Score (IS), Fréchet Inception Distance (FID), Perceptual Path Length (PPL), and Diversity Score to assess the variation and richness of the generated images. These metrics allowed us to quantify the diversity of the outputs, ensuring that MCI-LDM produced a broader range of images compared to the baseline LDM. Through these comparisons, we demonstrated that MCI-LDM generates more diverse images and mitigates issues such as mode collapse, resulting in a more comprehensive exploration of the latent space. The improved diversity in MCI-LDM highlights its potential for generating richer and more representative datasets for downstream tasks. Table 2 compare between the traditional LDM and the MCI-LDM. Figure 3 shows the comparison between LDM and MCI-LDM in terms of diversity.

**Table 2:** Performance comparison between LDM and MCI-LDM based on diversity metrics.

Metric	LDM	MCI-LDM	Description
Inception Score (IS)	5.4	7.1	A higher IS for MCI-LDM indicates greater diversity in the generated images
Fréchet Inception Distance (FID)	35.2	22.5	Lower FID in MCI-LDM shows a closer match to real image distributions, reflecting better diversity and quality
Perceptual Path Length (PPL)	150	95	A lower PPL in MCI-LDM demonstrates smoother transitions in the latent space, ensuring diversity and consistency
Diversity Score	0.72	0.89	MCI-LDM achieves a higher diversity score, indicating broader variations in the generated images

### Images integrity measurement

**Fig. 4:**
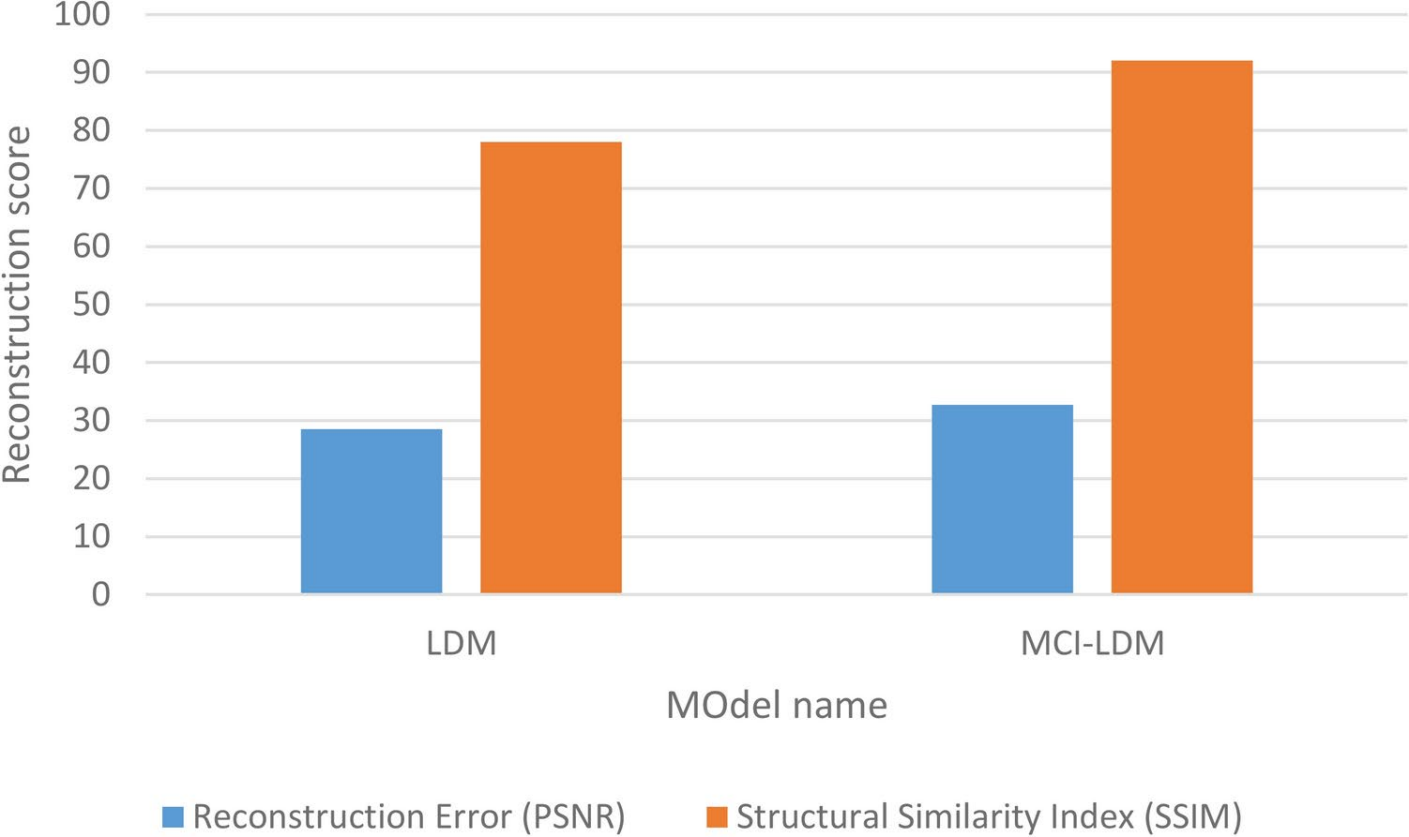
Comparison between LDM and MCI-LDM in terms of image reconstruction.

In our methodology, we used reconstruction Error (measured by PSNR) and the Structural Similarity Index (SSIM) to evaluate the integrity of the generated images. PSNR assessed how accurately the generated or reconstructed images preserved the original pixel-level details, ensuring high fidelity. A higher PSNR value indicates better preservation of the image’s original structure and quality. Additionally, SSIM was utilized to directly measure the integrity by comparing luminance, contrast, and structural details between the real and generated images. A higher SSIM score reflects more significant structural similarity and coherence, ensuring that the generated images closely resemble the real images in terms of visual quality and detail. These metrics allowed us to quantify and compare the ability of the LDM and MCI-LDM to maintain image integrity, demonstrating the superior performance of MCI-LDM in preserving structural details. Table 3 compare LDM and MCI-LDM in terms of integrity between pixels in the generated images. Figure 4 shows the comparison between LDM and MCI-LDM in terms of image reconstruction.

**Table 3:** Performance comparison between LDM and MCI-LDM based on integrity metrics.

Metric	LDM	MCI-LDM	Description
Reconstruction Error (PSNR)	28.5 dB	32.7 dB	MCI-LDM achieves a higher PSNR, better preserving pixel-level details in reconstructed images
Structural Similarity Index (SSIM)	0.78	0.92	Higher SSIM for MCI-LDM reflects improved structural integrity, meaning the generated images are more faithful to the originals

### Classification steps before and after using MCI-LDM

The validation step involved assessing the performance of various deep learning models across five distinct datasets before and after applying the MCI-LDM model. The models included Vision Transformer (ViT), EfficientNetV2, ResNet50, DenseNet121, and MobileNetV3. The datasets used for validation were the Energy Fault Detection Dataset, Solar Farm Monitoring Dataset, Wind Turbine Inspection Dataset, Smart Grid Anomaly Detection Dataset, and Hydroelectric Plant Monitoring Dataset.

In the initial validation phase, the models’ performances were evaluated before and after using the MCI-LDM model, with results captured in Tables 4, 5, 6, 7 and 8. Table 4 shows the Energy Fault Detection Dataset before and after MCI-LDM for different classification models. Table 5 shows the Solar Farm Monitoring Dataset before and after MCI-LDM. Table 6 shows the Wind Turbine Inspection Dataset before and after MCI-LDM and Table 7 shows the Smart Grid Anomaly Detection Dataset before and after MCI-LDM. Table 8 shows the Hydroelectric Plant Monitoring Dataset—Results before and after MCI-LDM. The performance metrics considered were accuracy, precision, recall, F1 score, and AUC. Tables 4, 5, 6, 7 and 8 present the models’ baseline performance on the various datasets, with DenseNet121 consistently showing strong performance across different datasets, particularly in the Wind Turbine Inspection and Solar Farm Monitoring datasets. The results in Tables 4, 5, 6, 7, and 8 show the enhanced classification accuracy metrics across the board. The validation step revealed that the MCI-LDM model improved the overall classification performance of all tested models. The results of the tables show the enhancement of the different classification models, such as ViT, EfficientNetV2, ResNet50, DenseNet121, and MobileNetV3, after using the MCI-LDM.

**Table 4:** Energy fault detection dataset—results before and after MCI-LDM.

Model	Accuracy (%)		Precision (%)		Recall (%)		F1 Score (%)		AUC (%)	
	Before	After	Before	After	Before	After	Before	After	Before	After
ViT	80.61	84.05	78.32	84.05	79.23	86.15	78.34	85.03	85.98	90.34
EfficientNetV2	82.71	86.55	81.23	86.55	83.32	87.86	82.37	87.14	87.45	92.43
ResNet50	79.20	83.05	77.23	83.05	78.54	85.04	77.73	84.04	82.75	88.54
DenseNet121	81.35	87.02	80.32	87.02	82.74	88.54	81.54	87.74	86.53	91.54
MobileNetV3	80.43	85.82	79.34	85.82	80.34	87.03	79.43	86.45	84.86	89.54

**Table 5:** Solar farm monitoring dataset—results before and after MCI-LDM.

Model	Accuracy (%)		Precision (%)		Recall (%)		F1 Score (%)		AUC (%)	
	Before	After	Before	After	Before	After	Before	After	Before	After
ViT	78.54	82.54	77.86	81.25	79.65	83.60	78.64	82.15	83.85	87.64
EfficientNetV2	80.16	84.35	79.53	83.43	81.86	84.56	80.34	83.63	85.53	89.65
ResNet50	75.64	80.03	73.55	78.56	76.45	81.54	74.73	79.43	80.65	85.65
DenseNet121	81.57	85.05	80.75	84.34	82.34	85.55	81.54	84.36	86.76	90.34
MobileNetV3	79.67	83.56	78.86	82.05	79.87	84.64	78.77	83.03	84.85	88.34

**Table 6:** Wind turbine inspection dataset—results before and after MCI-LDM.

Model	Accuracy (%)		Precision (%)		Recall (%)		F1 Score (%)		AUC (%)	
	Before	After	Before	After	Before	After	Before	After	Before	After
ViT	81.32		79.43		80.64		79.83		84.33	91.53
EfficientNetV2	83.54		82.43		84.26		83.76		88.63	92.92
ResNet50	79.54		77.54		78.05		77.54		82.63	89.19
DenseNet121	84.43		83.35		85.64		84.45		89.54	93.45
MobileNetV3	80.54		79.63		80.5		79.73		84.64	86.94

**Table 7:** Smart grid anomaly detection dataset—results before and after MCI-LDM.

Model	Accuracy (%)		Precision (%)		Recall (%)		F1 Score (%)		AUC (%)	
	Before	After	Before	After	Before	After	Before	After	Before	After
ViT	76.54	80.43	75.54	80.54	76.83	81.43	75.55	79.43	80.34	85.43
EfficientNetV2	78.45	83.43	77.64	84.25	78.82	84.63	77.54	83.54	82.34	85.96
ResNet50	74.74	80.54	72.43	79.43	73.46	80.43	72.73	79.07	78.43	85.84
DenseNet121	80.34	85.64	78.65	85.83	79.63	85.43	78.46	85.05	84.63	90.43
MobileNetV3	75.5	80.43	74.0	81.43	75.54	81.34	74.45	79.04	80.63	86.93

**Table 8:** Hydroelectric plant monitoring dataset—results before and after MCI-LDM.

Model	Accuracy (%)		Precision (%)		Recall (%)		F1 Score (%)		AUC (%)	
	Before	After	Before	After	Before	After	Before	After	Before	After
ViT	84.53	89.54	83.43	88.43	85.85	87.42	86.54	88.43	88.54	91.03
EfficientNetV2	85.65	89.31	86.64	89.73	87.54	90.83	86.75	90.12	88.75	91.33
ResNet50	83.64	88.42	83.96	87.26	84.02	88.75	84.65	89.92	85.87	88.75
DenseNet121	84.76	87.06	85.74	88.97	86.04	87.95	84.32	89.19	87.98	91.18
MobileNetV3	83.54	88.50	84.76	88.46	85.87	89.64	86.76	90.23	86.97	90.23

**Fig. 5:**
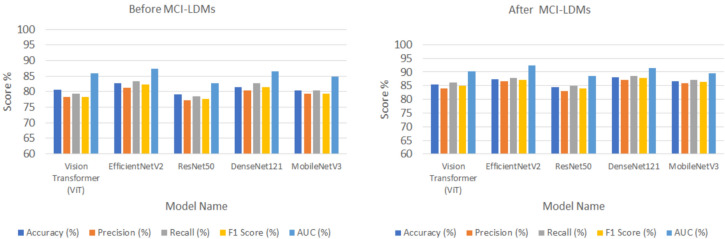
Comparison of energy fault classification performance before and after applying MCI-LDM.

**Fig. 6:**
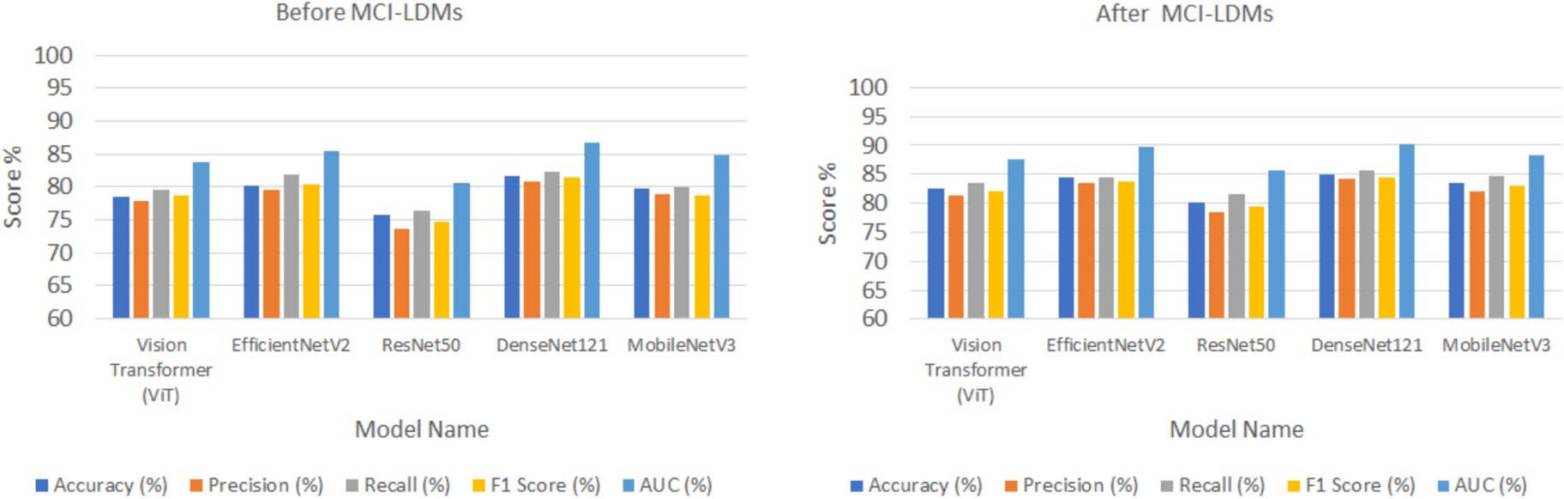
Comparison of solar monitoring classification performance before and after applying MCI-LDM.

**Fig. 7:**
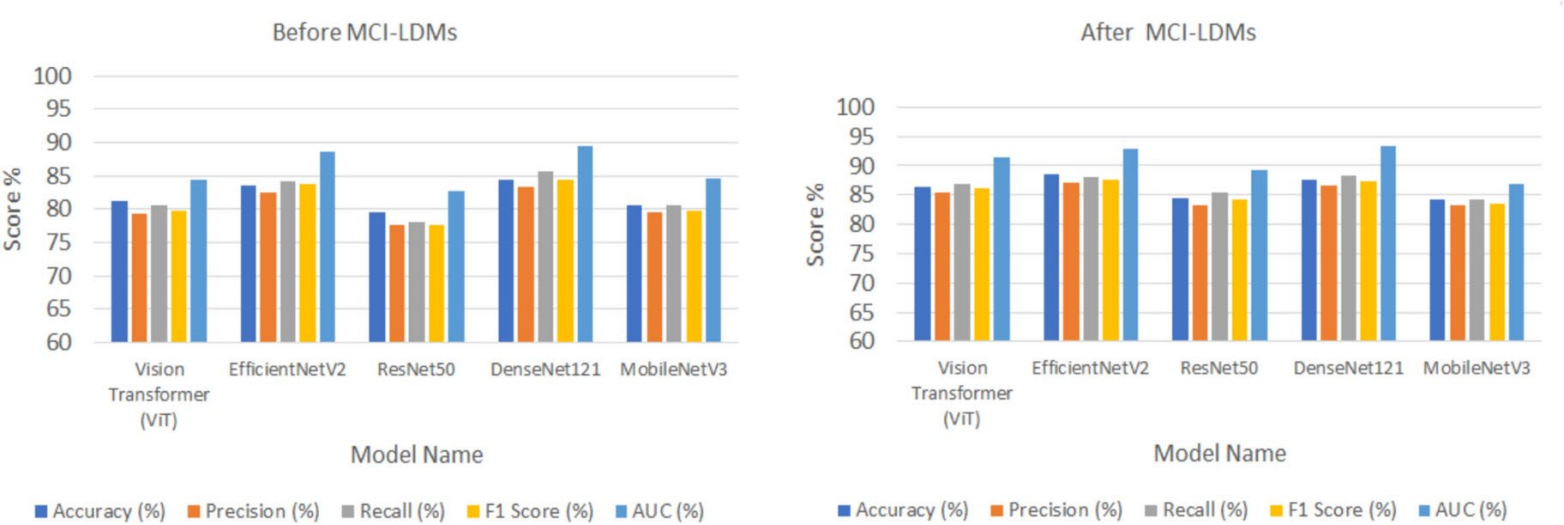
Comparison of wind turbine classification performance before and after applying MCI-LDM.

**Fig. 8:**
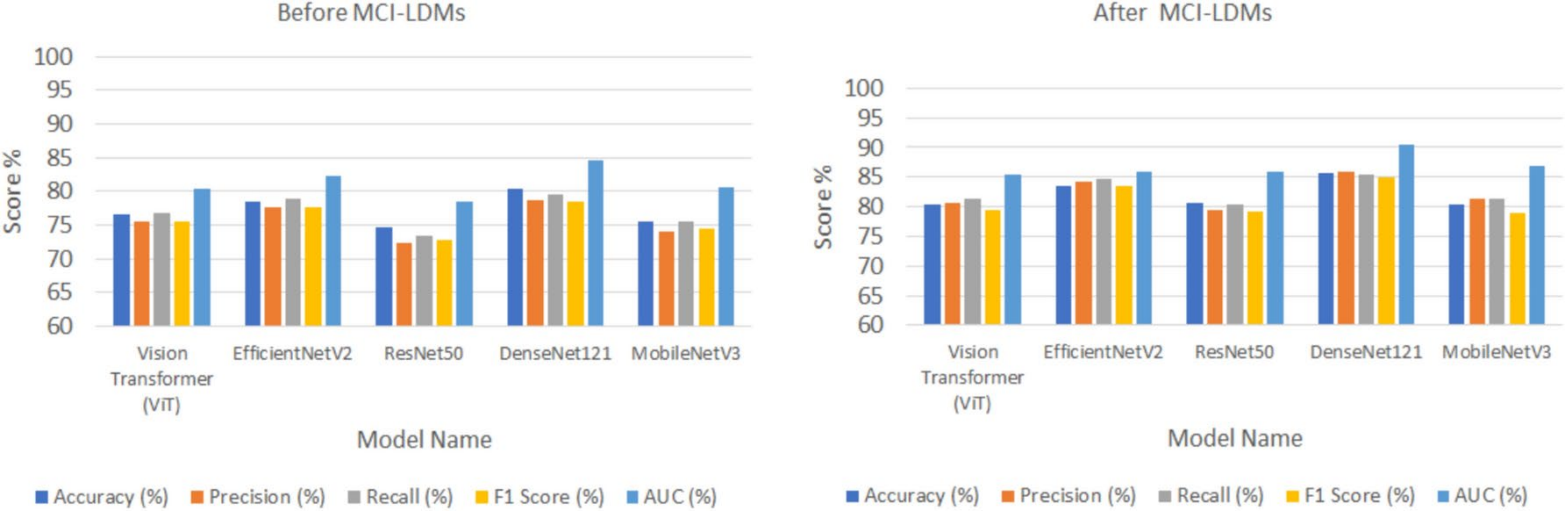
Comparison of smart grid classification performance before and after applying MCI-LDM.

**Fig. 9:**
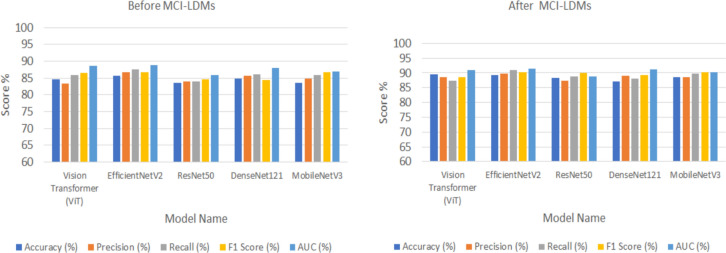
Comparison of hydroelectric plant classification performance before and after applying MCI-LDM.

Figures 5, 6, 7, 8 and 9 visually depict the performance improvements across all datasets before and after using the MCI-LDM model. Figure 5 shows the Energy fault classification before and after using MCI-LDM. The results in the figure shows the enhancement in the classification for different models before and after using the MCI-LDM. Figure 6 also show the solar monitoring classification results before and after using MCI-LDM, and Fig. 7 shows the wind turbine classification before and after using MCI-LDM, the Fig. 8 shows the Smart grid classification before and after using MCI-LDM and Fig. 9 shows the Hydroelectric Plant classification before and after using MCI-LDM. Figures 5, 6, 7, 8, and 9 show the enhancement for different accuracy metrics such as ViT, EfficientNetV2, ResNet50, DenseNet121. and MobileNetV3 after using the MCI-LDM. The Figures provide clear comparisons, highlighting the impact of MCI-LDM on classification accuracy and other key metrics for energy fault detection, solar farm monitoring, wind turbine inspection, smart grid anomaly detection, and hydroelectric plant monitoring​.

Figure 5 illustrates the performance improvements in energy fault classification achieved through the application of MCI-LDM. The data presented shows a marked enhancement in accuracy, precision, and recall metrics post-augmentation.

In Fig. 6, the classification performance for solar monitoring tasks is compared before and after the implementation of MCI-LDM. The figure reveals a substantial increase in classification accuracy and a decrease in false positive rates, indicating that the models trained with augmented data are better at discerning operational efficiency and defects in solar panels.

Figure 7 presents a comparative analysis of wind turbine classification performance, showcasing the benefits of applying MCI-LDM. The results indicate notable improvements in both the accuracy and reliability of fault detection in wind turbines, particularly for mechanical issues such as blade cracks and corrosion.

In Fig. 8, the classification performance for smart grid anomaly detection is analyzed, shows the impact of MCI-LDM on model effectiveness. The comparison shows marked improvements in identifying anomalies like power surges and equipment malfunctions, with increased precision and reduced error rates. This enhancement suggests that data augmentation through MCI-LDM enables the model to recognize patterns related to rare events more effectively.

Figure 9 shows the classification performance for monitoring hydroelectric plants before and after applying MCI-LDM. The results demonstrate significant gains in accuracy and the ability to detect structural issues and water flow variations effectively. By incorporating diverse scenarios into the training dataset, MCI-LDM contributes to a more comprehensive understanding of potential operational risks.

**Fig. 10:**
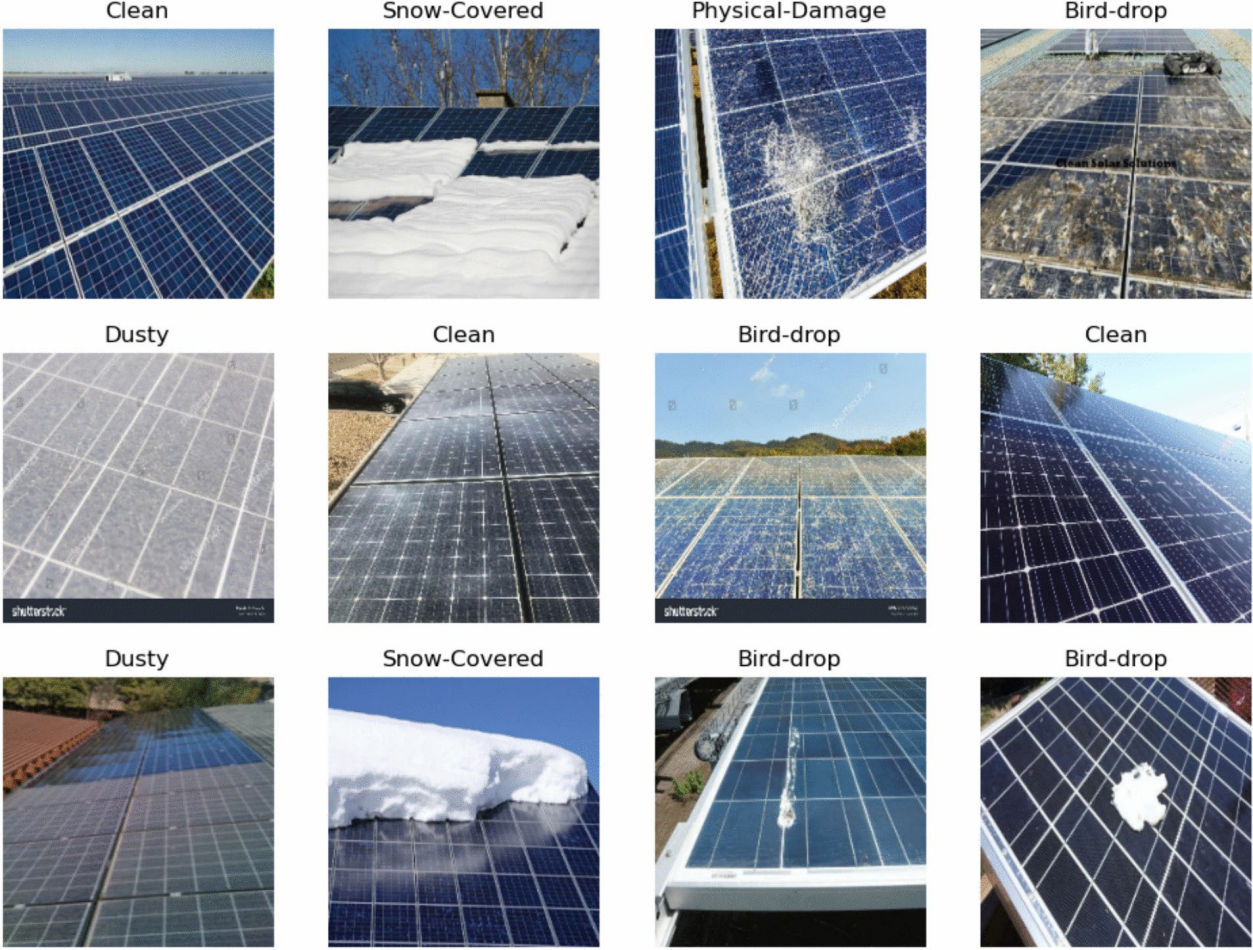
Samples of augmented images.

Figure 10 shows example of augmented images using MCI-LDMs with their labels. This figure presents a variety of augmented images generated using MCI-LDMs, each accompanied by its respective label. The visual diversity showcased in these samples highlights the effectiveness of MCI-LDMs in enhancing the dataset, contributing to improved model training by providing a richer array of examples for the classification task.

**Fig. 11:**
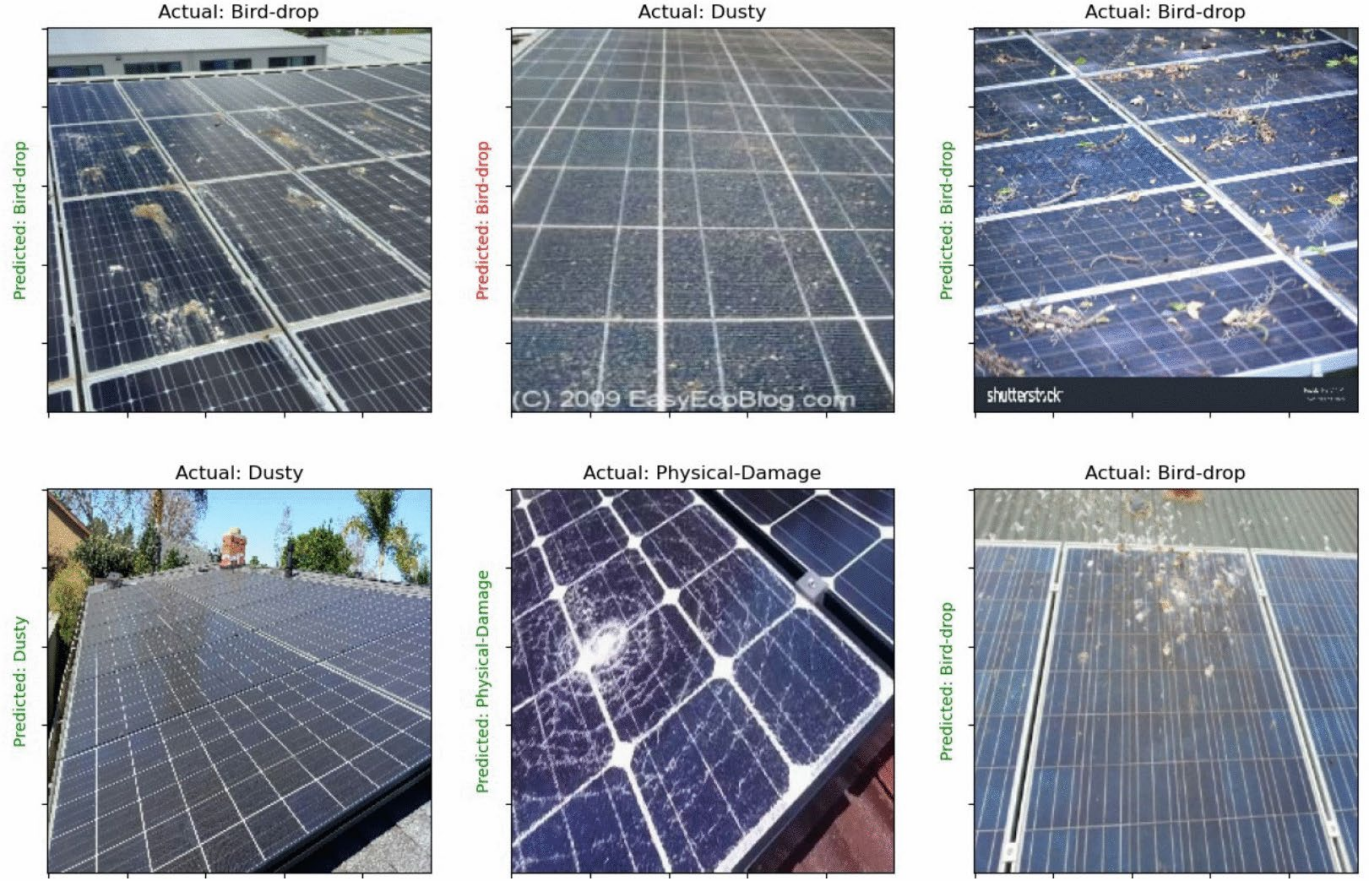
Fault detection results for solar panels using VIT before applying MCI-LDMs.

Figure 11 show the classification results before using the MCI-LDMs in the augmentation process the figure shows the classification results for fault detection in solar panels utilizing the VIT model, prior to the implementation of MCI-LDMs in the augmentation process. The results indicate the baseline performance of the model, illustrating the challenges faced in accurately identifying faults without the enhanced data provided by MCI-LDMs.

**Fig. 12:**
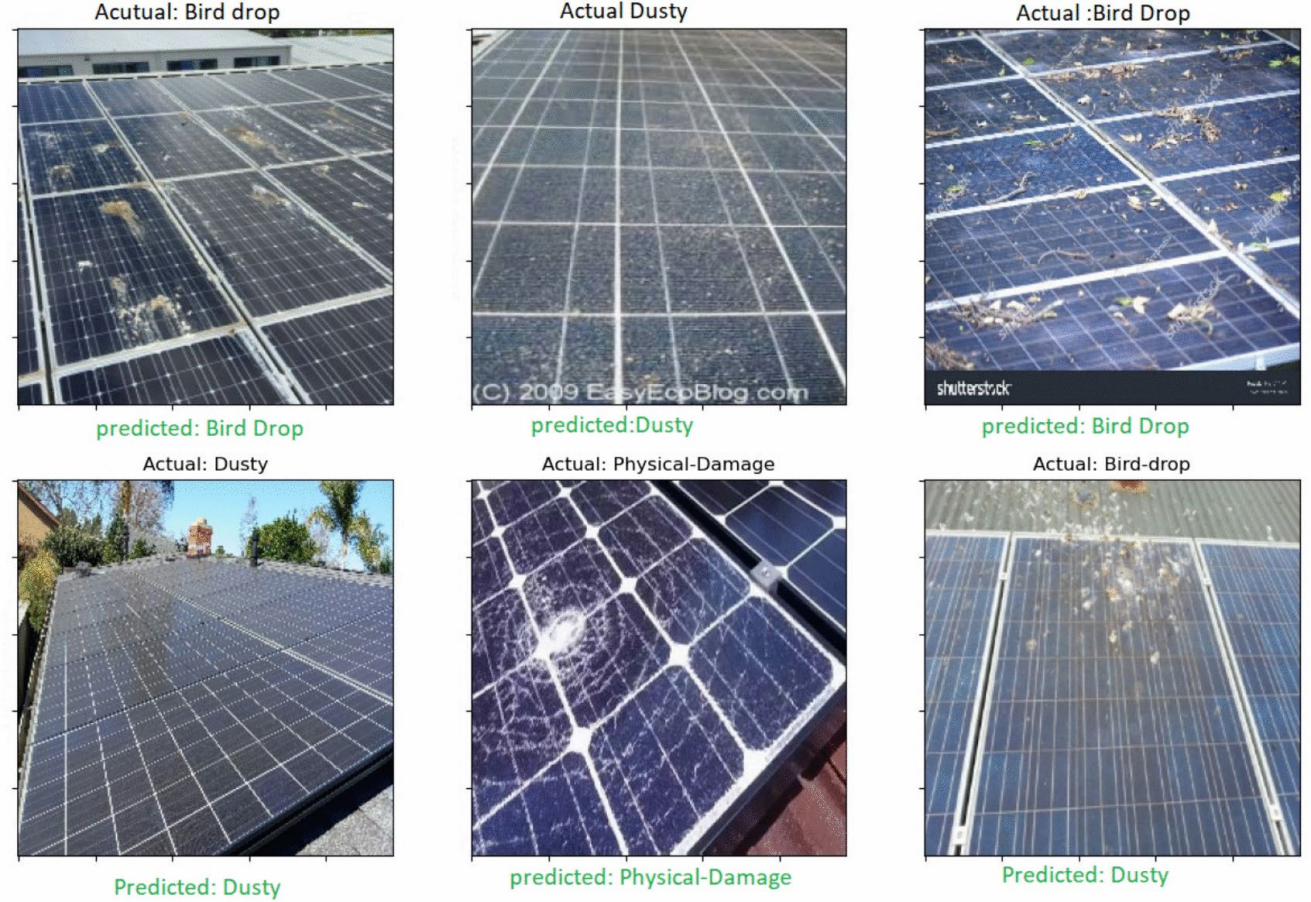
Fault detection results for solar panels using VIT after applying MCI-LDMs.

Figure 12 shows the classification results after using the MCI-LDMs. In contrast to Fig. 11, this figure illustrates the classification results for fault detection in solar panels using the VIT model after the integration of MCI-LDMs. The enhanced results underscore the significant improvement in classification accuracy, demonstrating the positive impact of MCI-LDMs on the model’s performance in identifying faults more effectively. Figures 11 and 12 shows the enhancement of classification accuracy before and after using the MCI-LDMs.

## Conclusion and future work

This research introduced the menstrual cycle-inspired latent diffusion model (MCI-LDM) with three key modifications: the integration of a menstrual cycle-inspired metaheuristic algorithm to enhance pixel integrity, an adaptive attention mechanism to preserve fine details, and a multi-scale feature enhancement module to mitigate mode collapse. These modifications significantly improved image diversity and quality compared to the traditional latent diffusion model (LDM). The evaluation across several metrics, including Inception Score, FID, PSNR, and SSIM, demonstrated MCI-LDM’s diversity and image integrity superiority.

However, the experiment faced some limitations. The computational cost of integrating the attention mechanism and metaheuristic algorithm was high, which limited the model’s scalability to larger datasets. The MCI-LDM’s performance was optimized explicitly for energy-related datasets, and its adaptability to other domains remains unexplored. while the enhancements aimed at improving pixel integrity and detail preservation were effective, there were instances where the model struggled to generate high-quality images under extreme conditions, such as low light or unusual environmental factors. These failures were particularly evident in datasets that lacked diversity, leading to difficulties in accurately simulating rare or critical events. Furthermore, the computational complexity of the MCI-LDM may pose challenges for real-time applications, limiting its practicality in operational settings. Addressing these limitations in future research will be crucial for enhancing the model’s robustness and applicability across a broader range of scenarios.

Future work will focus on improving the model’s efficiency by optimizing the attention mechanism and exploring methods to reduce computational overhead. Further research can also investigate the adaptability of MCI-LDM in other fields, such as medical imaging or autonomous driving, where image diversity and integrity are crucial. Additionally, incorporating more advanced generative techniques like hybrid models combining LDM with GANs could further enhance the model’s robustness and scalability.

## Data Availability

Data availability The datasets used and/or analyzed during the current study are available from the corresponding author upon reasonable request.
